# Supplementary material for: An efficient approach for low latency processing in stream data
Source: PeerJ Comput Sci. 2021 Mar 10;7:e426. doi: 10.7717/peerj-cs.426 (PMC7959664; doi:10.7717/peerj-cs.426)
Supplement: Supplemental Information 1 — The cloud script and related dataset [file peerj-cs-07-426-s001.zip › Code/apache-beam-2.4.0/sdks/java/io/tika/src/test/resources/valid/apache-beam-tika-pdf.zip!/apache-beam-tika.pdf]

Combining

Apache Beam

and

Apache Tika

can help to ingest

the content from the files

in most known formats.
