# Supplementary material for: An efficient approach for low latency processing in stream data
Source: PeerJ Comput Sci. 2021 Mar 10;7:e426. doi: 10.7717/peerj-cs.426 (PMC7959664; doi:10.7717/peerj-cs.426)
Supplement: Supplemental Information 1 — The cloud script and related dataset [file peerj-cs-07-426-s001.zip › Code/apache-beam-2.4.0/sdks/java/javadoc/overview.html]

Apache Beam Java SDK


The Apache Beam SDK for Java provides a simple and elegant
programming model to express your data processing pipelines;
see the Apache Beam website
for more information and getting started instructions.

The easiest way to use the Apache Beam SDK for Java is via
one of the released artifacts from the
Maven Central Repository.

Version numbers use the form *major*.*minor*.*incremental*
and are incremented as follows:

- major version for incompatible API changes
- minor version for new functionality added in a backward-compatible manner
- incremental version for forward-compatible bug fixes

Please note that APIs marked
{@link org.apache.beam.sdk.annotations.Experimental @Experimental}
may change at any point and are not guaranteed to remain compatible across versions.
