# Supplementary material for: An efficient approach for low latency processing in stream data
Source: PeerJ Comput Sci. 2021 Mar 10;7:e426. doi: 10.7717/peerj-cs.426 (PMC7959664; doi:10.7717/peerj-cs.426)
Supplement: Supplemental Information 1 — The cloud script and related dataset [file peerj-cs-07-426-s001.zip › Code/apache-beam-2.4.0/sdks/python/apache_beam/examples/complete/Stock_Prediction/Step-By-Step.docx]

1) First I have executed word count.
